# SABA use as an indicator for asthma exacerbation risk: an observational cohort study (SABINA Canada)

**DOI:** 10.1183/23120541.00140-2022

**Published:** 2022-09-26

**Authors:** Stephen G. Noorduyn, Christina Qian, Karissa M. Johnston, Mena Soliman, Manisha Talukdar, Brandie L. Walker, Paul Hernandez, Erika Penz

**Affiliations:** 1 Dept of Medical Affairs, AstraZeneca Canada, Mississauga, Canada; 2 Dept of Health Research Methods, Evidence & Impact, McMaster University, Hamilton, Canada; 3 Broadstreet HEOR, Vancouver, Canada; 4 Memorial University, St John's, Canada; 5 Division of Respirology, Dept of Medicine, University of Calgary, Calgary, Canada; 6 Division of Respirology, Dept of Medicine, Dalhousie University, Halifax, Canada; 7 Division of Respirology, Critical Care and Sleep Medicine, University of Saskatchewan, Saskatoon, Canada

## Abstract

**Background:**

Patients with asthma use short-acting β-agonists (SABA) to relieve symptoms but SABA alone does not treat underlying inflammation. Thus, over-reliance on SABA may result in poor asthma control and negative health outcomes.

**Objective:**

To describe use of SABA and characterise the relationship with severe exacerbations in the Canadian provinces of Nova Scotia (NS) and Alberta (AB).

**Methods:**

In this longitudinal Canadian SABA In Asthma (SABINA) study, patients with an asthma diagnosis were identified between 2016 and 2020 within two provincial administrative datasets (Health Data Nova Scotia and Alberta Health Services). All patients were followed for ≥24 months, with the first 12 months used to measure baseline asthma severity. Medication use and the relationship of SABA overuse (three or more canisters per year) with severe asthma exacerbations were characterised descriptively and *via* regression analysis.

**Results:**

A total of 115 478 patients were identified (NS: n=8034; AB: n=107 444). SABA overuse was substantial across both provinces (NS: 39.4%; AB: 28.0%) and across all baseline disease severity categories. Patients in NS with SABA overuse had a mean±sd annual rate of 0.46±1.11 exacerbations, compared to 0.30±1.36 for those using fewer than three canisters of SABA. Patients in AB had mean±sd exacerbation rates of 0.31±0.86 and 0.17±0.62, respectively. The adjusted risk of severe exacerbation was associated with SABA overuse (NS: incidence ratio rate 1.36, 95% CI 1.18–1.56; AB: incidence ratio rate 1.32, 95% CI 1.27–1.38).

**Conclusion:**

This study supports recent updates to Canadian Thoracic Society and Global Initiative for Asthma guidelines for asthma care. SABA overuse is associated with increased risk of severe exacerbations and can be used to identify patients at a higher risk for severe exacerbations.

## Abstract 2

**This longitudinal study supports the recent updates to the CTS and GINA guidelines for asthma care. SABA utilisation can be used to identify patients at higher risk of severe exacerbation, regardless of disease severity.** https://bit.ly/3Nd3USz

## Introduction

Asthma is a chronic disease affecting nearly 10% of Canadians and nearly 65 000 asthma exacerbations occur each year [1–4]. It is estimated that >50% of patients with asthma continue to experience exacerbations and regular symptoms throughout their daily life [5, 6]. During periods of poor control, rescue inhalers containing short-acting β-agonists (SABA) are commonly used to relieve acute symptoms, despite leaving the underlying airway inflammation untreated. However, the overuse of SABA (three or more canisters per year) is associated with asthma-related hospital admissions, emergency department visits and an overall increase in healthcare costs [7–20]. Previous studies have found that excessive use (12 or more annual canisters) is even associated with an increased risk of death [19, 21–23].

In 2017, O’Byrne *et al.* [23] highlighted paradoxes of asthma management. At the time, SABA alone was recommended for patients categorised as Global Initiative for Asthma (GINA) Step One even though asthma is a chronic airway inflammatory disease. O’Byrne *et al.* [23] noted that when patients progress to GINA Step Two, they initiate regular inhaled corticosteroid (ICS) therapy and are asked to minimise the as-needed SABA upon which they previously relied. This change requires a mindset shift, in which pharmacotherapy is no longer autonomous, symptom-based and associated with fast-acting relief. Instead, patients are expected to adhere to daily or twice daily doses of ICS-based maintenance therapy regardless of their symptoms. However, this means patients do not experience the rapid symptom relief associated with their previous treatment regimen. As a result, they may undervalue adherence to maintenance therapy and simply increase SABA use when symptoms arise. While there have been some targeted studies of the use of SABA in Canada [24], little has been done to quantify the scale of overuse and the impact on patient outcomes.

The SABA in Asthma (SABINA) study is a global programme to evaluate utilisation and clinical outcomes related to SABA use in asthma; this study is the Canadian contribution to that programme [15, 25]. The objectives of this paper were to describe the use of SABA and characterise the relationship with severe exacerbations in the two Canadian provinces of Nova Scotia (NS) and Alberta (AB).

## Materials and methods

### Study design and data sources

This study is a retrolective cohort study [26] using administrative claims datasets from Health Data Nova Scotia (HDNS) [27] and Alberta Health Services (AHS) [28]. These are provincial health data repositories for nearly one million individuals in NS and over four million individuals in AB. Both datasets include demographics (*e.g.* sex, date of birth) and administrative claims information on publicly reimbursed health resource use (*e.g.* medication dispensation, physician visits) and outcomes (*e.g.* hospital admissions, mortality). Data from both provinces were analysed and reported separately to account for variability in content and coding as well as recognition of data security and privacy requirements from both health systems [29].

### Study period definitions

This study used the most recent available data at the time of extraction: from October 2016 to March 2019 for NS, and April 2016 to March 2020 for AB. Three time periods were defined within the study. The baseline period was used to assess case definition and to characterise baseline comorbidity, asthma disease severity, the frequency of asthma-related prescription claims (supplementary table S1), physician visits and exacerbations in the 12 months following the diagnosis meeting the case definition. The index date occurred at the end of the baseline period, or 365 days after the first date on which the patient had an eligible diagnosis, and was used to summarise the age and sex of the patient (supplementary figure S1). The remainder of the follow-up was designated the study period and was used to measure study exposures and outcomes. The study period began at index and ended at the time of censoring, the first occurrence of patients moving out of the province, death or by the end of the study in March 2019 in NS or March 2020 in AB.

### Eligibility criteria

Data were linked at the individual level to identify a patient cohort for analysis. Patients who were diagnosed with asthma and had at least two consecutive years of follow-up data were eligible for inclusion [30]. We used a validated case definition for asthma within Canadian administrative datasets (two or more physician visits in a 2-year period or one or more hospital admission with a diagnosis of asthma (International Classification of Disease (ICD) 9th revision, Clinical Modification 493 or ICD 10th revision, Canadian version J45) [31]. Patients were included in the study population if they were ≥12 years old with active records (or known reason for inactivity, *e.g.* death) throughout the study period. Patients were excluded if they were ≥35 years with a diagnosis of chronic obstructive pulmonary disease, if they had <12 months of data available prior to the index date, if they did not have active asthma (no records of asthma diagnosis or treatment following the index date) or if they had severe asthma and were using biological therapies for disease management (supplementary table S2).

### Baseline asthma severity definitions

Asthma severity in the baseline period was determined using average daily dose for ICS (low, medium, high dose) as per the Canadian Thoracic Society (CTS) guidelines [32]. Baseline asthma severity was also categorised into GINA Steps 1–5 per the 2018 GINA recommendations (supplementary material) [33]. Under the CTS definition, patients meeting the case definition but not using any ICS or SABA in the baseline period were included but presented as a distinct subgroup (“mild, no prescription”) throughout the analysis.

### Study variables

The exposures of interest included use (*i.e.* prescriptions dispensed) of ICS and SABA (see supplementary table S1 for included drug identification numbers). ICS in this study refers to all ICS-containing products, *i.e.* including both ICS monotherapy and ICS/long-acting β-agonist (LABA) combination therapy. SABA dispensations were standardised to a 150-dose canister (for consistency with the global SABINA programme [19, 25]) and annualised over the study period. SABA overuse was defined as three or more canisters per year [17], and 12 or more canisters used per year was considered excessive use [34]. An exploratory analysis using the average number of SABA doses per week was also calculated, defining three or more doses of SABA as “beyond the suggested use”, and 10 or more doses as overuse, as per the CTS guidelines [34]. This alternative quantification permits broader interpretation of the study result in Canadian practice. All dosage calculations assumed that all dispensed medication was completely utilised over the study period [25]. Details of how dispensed prescription medication was used to estimate annual use and weekly use are described in the supplementary material.

Outcomes of interest included severe asthma exacerbations and all-cause mortality. Severe exacerbations were defined by dispensation of short-course (≤10 days) oral corticosteroids (OCS) [35] or by hospital admission or emergency department visit with a primary complaint of asthma (supplementary table S3).

### Ethics approval

This study received ethical approval from the Health Sciences Research Ethics Boards at Dalhousie University (REB2019-4959/November 29, 2019) and the Health Research Ethics Board of Alberta (REB20-0010/March 5, 2020) for secondary use of information for research. All individual identifiable information was anonymised prior to data analysis and small number subgroups were not reported to prevent identification.

### Statistical analysis

Patient demographics and baseline characteristics including age, sex, comorbidity burden summarised using the Elixhauser comorbidity score [36] and health-seeking events are presented descriptively. Post-index medication use and exacerbations are also descriptively summarised for the overall cohort and stratified by baseline disease severity and SABA use over the study period. The association between SABA overuse and the number of severe exacerbations was further evaluated by means of a negative binomial regression adjusting for demographic and clinical characteristics. This analysis was limited to the first year of the study period to standardise follow-up available across the two patient populations. The unadjusted and adjusted association between SABA overuse and rate of severe exacerbations was reported using incidence rate ratio (IRR) and 95% confidence intervals, overall and stratified by GINA Step. Subgroup analysis among patients using six or more canisters of ICS (that is, adherent to 50% or more days of maintenance annually) was also performed to stratify patients by maintenance therapy compliance. It should be noted that the number of canisters was used as a measure of compliance rather than the proportion of days covered because it is a more reliable measure for compliance in Canadian claims data [37]. All analyses were limited to available data and no missing data were imputed. All results are reported separately by provincial data source.

## Results

A total of 115 478 patients was included in the study (NS: n=8034; AB: n=107 444) (supplementary figure S2). At index, patients from NS had slightly higher mean±sd age (NS: 43.4±18.3 years; AB: 39.7±17.1 years) and Elixhauser comorbidity score (NS: 2.01±1.27; AB: 1.21±1.07) than patients from AB (table 1). Commonly observed comorbid diagnoses included anxiety (NS: 16.2%; AB: 27.6%), diabetes (NS: 7.5%; AB: 1.8%), gastro-oesophageal reflux (NS: 4.2%; AB: 0.9%), pneumonia (NS: 4.4%; AB: 4.5%) and bone fractures (NS: 2.4%; AB: 6.6%; supplementary table S4).

**TABLE 1:** Patient demographics at baseline^#^ among patients with asthma in Nova Scotia and Alberta

	**Primary study cohort**	
	**Nova Scotia**	**Alberta**
**Subjects, n**	8034	107 444
**Follow-up, median (IQR) (days)**	442 (386–490)	730 (730–730)
**Sex, n (%)**		
Male	3171 (39.5)	46 910 (43.7)
Female	4863 (60.5)	60 534 (56.3)
**Age (years)**		
Mean±sd	43.4±18.3	39.7±17.1
Median (IQR)	43.0 (29.0–57.0)	38.0 (26.0–52.0)
**Elixhauser comorbidity score, mean±sd**	2.01±1.27	1.21±1.07
**Asthma severity, n (%)**		
Mild asthma	6694 (83.3)	91 189 (84.9)
Mild, no prescription	880 (11.0)	29 353 (27.3)
Mild, excluding those with no prescription	5814 (72.4)	61 836 (57.6)
Moderate asthma	998 (12.4)	10 412 (9.7)
Severe asthma, no biological treatment	342 (4.3)	5843 (5.4)
**All outpatient visits**		
Mean±sd	1.9±1.3	1.6±2.8
Median (IQR)	2.0 (1.0–2.0)	1.0 (0.0–2.0)
**Asthma-specific prescriptions**		
Mean±sd	5.4±5.1	5.0±4.5
Median (IQR)	4.0 (2.0–8.0)	4.0 (2.0–7.0)
**Exacerbations,** **^¶^ n (%)**		
ED visits		
0	7922 (98.6)	102 098 (95.0)
1	100 (1.2)	4341 (4.0)
2	11 (0.1)	709 (0.7)
3	<5 (0)	176 (0.2)
4+	<5 (0)	62 (0.1)
Hospitalisations (excluding ED visits)		
0	8033 (100)	106 303 (98.9)
1	<5 (0)	1071 (1.0)
2	<5 (0)	58 (0.1)
3	<5 (0)	11 (0.0)
4+	<5 (0)	<5 (0)
Prescriptions for OCS		
0	6171 (76.8)	93 070 (86.6)
1	1210 (15.1)	10 054 (9.4)
2	375 (4.7)	2501 (2.3)
3	129 (1.6)	882 (0.8)
4+	149 (1.9)	937 (0.9)
Severe exacerbations**^+^**		
0	6149 (76.5)	95 030 (88.4)
1	1218 (15.2)	8855 (8.2)
2	381 (4.7)	2150 (2.0)
3	137 (1.7)	699 (0.7)
4+	149 (1.9)	710 (0.7)

Cohorts include patients with an asthma diagnosis identified between 2016 and 2020 within two provincial administrative datasets (Health Data Nova Scotia and Alberta Health Services). ED: emergency department; OCS: oral corticosteroid. ^#^: 1 year following diagnosis; ^¶^: unique visit dates and/or admission dates; ^+^: if more than one of the events (hospitalisation or ED with primary diagnosis of asthma, or oral corticosteroid) occurred within a 2-week window, this was counted as one exacerbation.

### Medication use

In the baseline period, >80% of both cohorts received no or low-dose ICS, meeting the case definition of mild asthma. Approximately 11% (NS) and 27% (AB) of patients had no asthma-related prescription use in the baseline period. In both cohorts, patients filled a mean±sd of 5±5 asthma medication prescriptions, and had 2±1.3 and 2±2.8 outpatient visits in the baseline period for NS and AB, respectively (table 1).

**FIGURE 1:**
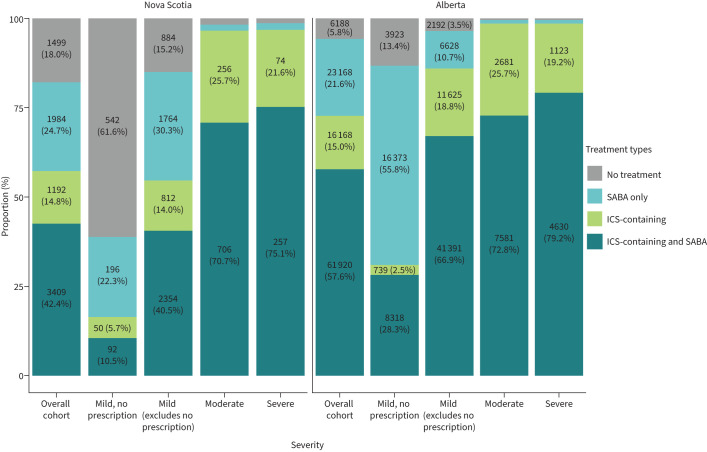
Overall treatment patterns over the study period, stratified by baseline severity. SABA: short-acting β-agonists; ICS: inhaled corticosteroids.

During the study period, most patients in both provinces received ICS-based maintenance therapy combined with a SABA rescue inhaler (figure 1). In patients who did not fill any prescription during the baseline year, the majority of patients in NS (n=542, 61.6%) also did not receive ICS-containing or SABA medications during the entire study period. Conversely, most patients in AB within this group were dispensed either ICS-containing or SABA medication during the study period (55.8% with SABA monotherapy, 2.5% with ICS-containing therapy and 28.3% with ICS-containing and SABA). Further trends in use of ICS and SABA during the study period are available in table 2 and supplementary table S5, stratified by baseline disease severity defined using CTS and GINA, respectively.

**TABLE 2:** ICS and SABA use during the study period by baseline disease severity

		**Nova Scotia**					**Alberta**			
		**Baseline asthma severity**					**Baseline asthma severity**			
	**Overall** ^#^	**Mild, no prescription** ^¶^	**Mild, with prescription** ^+^	**Moderate** ^§^	**Severe** ^ƒ^	**Overall**^#^	**Mild, no prescription** ^¶^	**Mild, with prescription** ^+^	**Moderate** ^§^	**Severe** ^ƒ^
**Subjects, n**	8034	880	5814	998	342	107 444	29 353	61 836	10 412	5843
**ICS dose**										
No ICS	3301 (41.1)	738 (83.9)	2539 (43.7)	17 (1.7)	7 (2.0)	29 356 (27.3)	20 296 (69.1)	8820 (14.3)	150 (1.4)	90 (1.5)
Reduced	484 (6.0)			347 (34.8)	137 (40.1)	7320 (6.8)			4651 (44.7)	2669 (45.7)
Stable	3878 (48.3)		2960 (50.9)	582 (58.3)	198 (57.9)	66 150 (61.6)		49 197 (79.6)	4976 (47.8)	3084 (52.8)
Increased	371 (4.6)	142 (16.1)	315 (5.4)	52 (5.2)		4618 (4.3)	9057 (30.9)	3819 (6.2)	635 (6.1)	
**SABA use (canisters per year)**										
0	2641 (32.9)	592 (67.3)	1696 (29.2)	274 (27.5)	79 (23.1)	22 356 (20.8)	4662 (15.9)	13 817 (22.3)	2726 (26.2)	1151 (19.7)
1–2	2226 (27.7)	214 (24.3)	1686 (29)	245 (24.5)	81 (23.6)	55 056 (51.2)	17 148 (58.4)	31 613 (51.1)	4075 (39.1)	2220 (38.0)
3+	3167 (39.4)	74 (8.4)	2432 (41.8)	479 (48.0)	182 (53.2)	30 032 (28.0)	7543 (25.7)	16 406 (26.5)	3611 (34.7)	2472 (42.3)
12+	961 (12.0)	7 (0.8)	724 (12.5)	161 (16.1)	69 (20.2)	3624 (3.4)	1000 (3.4)	1715 (2.8)	495 (4.8)	414 (7.1)

Data presented as n (%), unless otherwise indicated. ICS: inhaled corticosteroids; SABA: short-acting β-agonists. ^#^: includes all patients in respective provinces; ^¶^: includes patients with no prescription dispensed in the baseline period; ^+^: includes patients with an average daily dose of ICS that is considered to be a low dose in the baseline period; ^§^: includes patients with an average daily dose of ICS that is considered to be a moderate dose in the baseline period; ^ƒ^: includes patients with an average daily dose of ICS that is considered to be a high dose in the baseline period.

**FIGURE 2:**
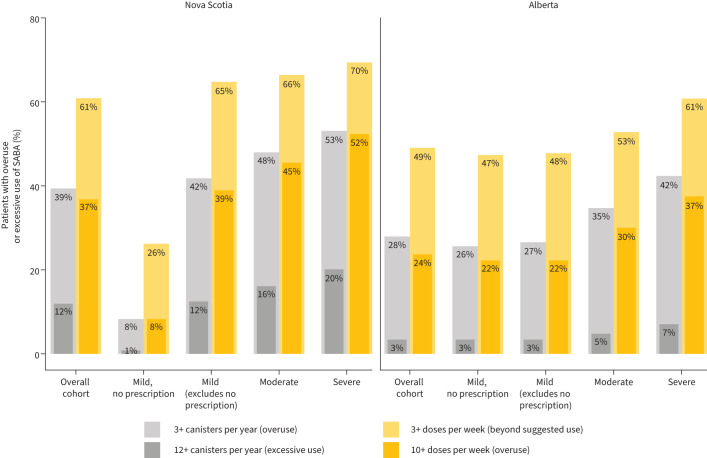
Overuse and excessive use of short-acting β-agonists (SABA) during the study period, stratified by baseline severity.

Throughout the study period, between 28% and 39% of patients met the definition of SABA overuse (NS: n=3167, 39.4%; AB: n=30 032, 28.0%) across all baseline disease severity categories. In both provinces, the percentage of patients overusing SABA was associated with baseline severity of disease. Excessive use of SABA was also higher in NS (n=961, 12.0%) than AB (n=3624, 3.4%) and this excessive use of SABA also increased with baseline severity of disease (table 2). In exploratory analyses using dose as the unit of measure, ∼50–60% of patients in both provinces used an average of at least three doses of SABA per week for the study period, indicating use beyond the suggested amount, and the proportion of patients using at least 10 doses per week was comparable to that observed for the overuse definition of three of more canisters per year (figure 2).

### Exacerbations

**FIGURE 3:**
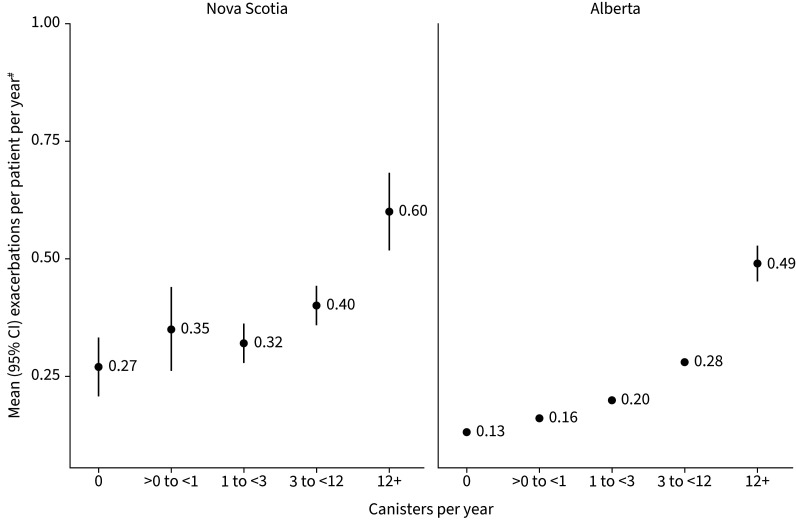
Number of severe exacerbations by short-acting β-agonists use during the study period. ^#^: if more than one event (hospitalisation or emergency department visit with primary diagnosis of asthma, or oral corticosteroid) occurred within a 2-week window, this was counted as one exacerbation (also included death)

**FIGURE 4:**
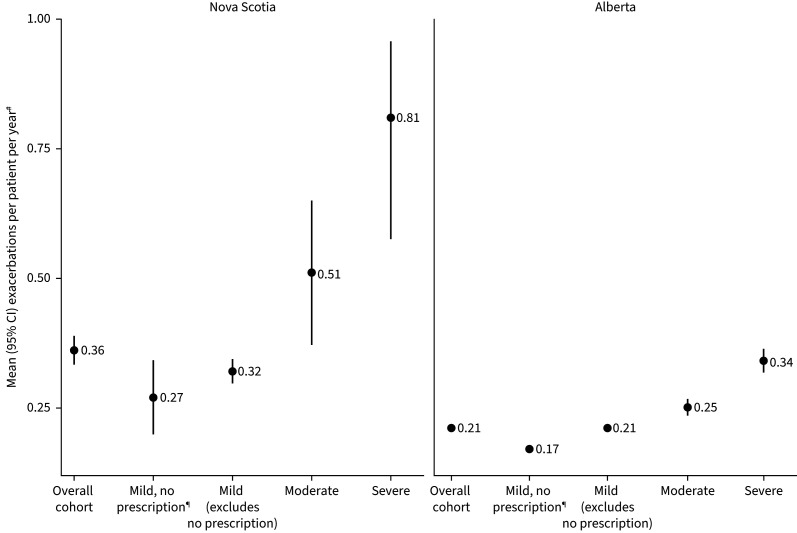
Number of severe exacerbations by baseline asthma severity. ^#^: if more than one event (hospitalisation or emergency department visit with primary diagnosis of asthma, or oral corticosteroid) occurred within a 2-week window, this was counted as one exacerbation (also included death); ^¶^: patients who did not have an inhaled corticosteroid or short-acting β-agonist prescription at anytime throughout the baseline period.

Between 10% and 25% of patients experienced a severe exacerbation during the baseline period (NS: 24.5%; AB: 11.6%; table 1). During the study period, few patients had any severe exacerbation (NS: 9.5%; AB: 7.9%; table 3). Annual rates of severe exacerbations increased with SABA use (figure 3) and baseline asthma severity (figure 4 and table 4), with estimates being consistently higher in the NS cohort (tables 3 and 4). Mean±sd estimates of annual exacerbations in NS increased from 0.30±1.36 among patients with appropriate use to 0.46±1.11 among those with SABA overuse and 0.60±1.31 among those with excessive use of SABA. In AB, annual estimates increased from 0.17±0.62 to 0.31±0.86 and 0.49±1.19, respectively. When stratifying by baseline disease severity, in NS, estimates increased from 0.27±1.09 among those with mild asthma (no prescriptions) to 0.81±2.23 among those with severe asthma. In AB, these estimates increased from 0.17±0.66 to 0.34±0.91. A similar trend was noted when baseline disease severity was classified using GINA Steps. Patients defined as GINA Step 5 demonstrated an annual rate of exacerbation six to nine times greater than found in patients of GINA Step 1 (supplementary table S6).

**TABLE 3:** Severe exacerbations by SABA use during study period

	**SABA canisters used during the study period in:**							
	**Nova Scotia**				**Alberta**			
	**Overall**	**<3**	**3+**	**12+**	**Overall**	**<3**	**3+**	**12+**
**Subjects, n**	8034	4867	3167	961	107 444	77 412	30 032	3624
**Annual exacerbations** **^#^**								
<1	7270 (90.5)	4508 (92.6)	2762 (87.2)	811 (84.4)	98 997 (92.1)	72 613 (93.8)	26 384 (87.9)	2952 (81.5)
1–2	391 (4.9)	192 (3.9)	199 (6.3)	59 (6.1)	6115 (5.7)	3578 (4.6)	2537 (8.4)	406 (11.2)
2+	373 (4.6)	167 (3.4)	206 (6.5)	91 (9.5)	2332 (2.2)	1221 (1.6)	1111 (3.7)	266 (7.3)
Mean±sd per patient	0.36±1.27	0.30±1.36	0.46±1.11	0.60±1.31	0.21±0.70	0.17±0.62	0.31±0.86	0.49±1.19

Data presented as n (%), unless otherwise indicated. SABA: short-acting β-agonists. ^#^: multiple events (hospitalisation or emergency department visit with primary diagnosis of asthma; oral corticosteroid dispensation; death) within a 2-week window were considered one exacerbation.

**TABLE 4:** Severe exacerbations by baseline disease severity

	**Baseline severity in:**							
	**Nova Scotia**				**Alberta**			
	**Mild, no prescription**	**Mild, with prescription**	**Moderate**	**Severe** **^¶^**	**Mild, no prescription**	**Mild, with prescription**	**Moderate**	**Severe** **^¶^**
**Subjects, n**	880	5814	998	342	29 353	61 836	10 412	5843
**Annual exacerbations** **^#^**								
<1	819 (93.1)	5305 (91.2)	871 (87.3)	274 (80.4)	27 607 (94.1)	56 931 (92.1)	9390 (90.2)	5069 (86.8)
1–2	30 (3.4)	277 (4.8)	61 (6.1)	23 (6.7)	1288 (4.4)	3609 (5.8)	710 (6.8)	508 (8.7)
2+	31 (3.5)	232 (4.0)	66 (6.6)	44 (12.9)	458 (1.6)	1296 (2.1)	312 (3.0)	266 (4.6)
Mean±sd per patient	0.27 (1.09)	0.32 (0.94)	0.51 (2.25)	0.81 (2.23)	0.17 (0.66)	0.21 (0.66)	0.25 (0.82)	0.34 (0.91)

Data presented as n (%), unless otherwise indicated. ^#^: multiple events (hospitalisation or emergency department visit with primary diagnosis of asthma; oral corticosteroid dispensation; death) within a 2-week window were considered one exacerbation; ^¶^: patients with severe asthma not using a biological therapy

**FIGURE 5:**
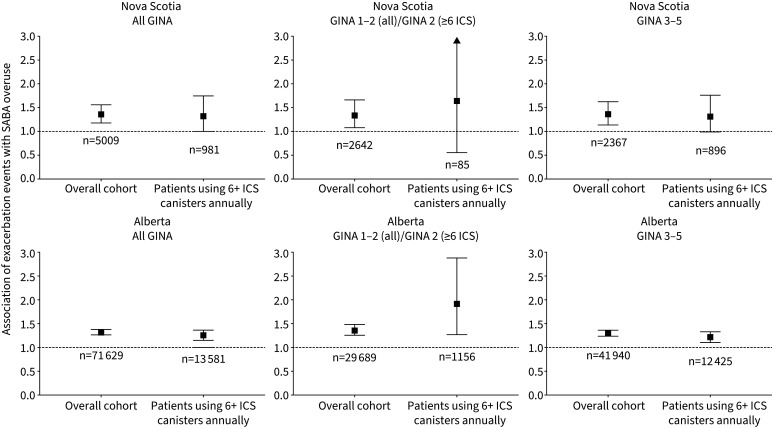
Adjusted incidence rate ratio for exacerbations associated with short-acting β-agonist (SABA) overuse (three or more canisters *versus* fewer than three canisters per year), stratified by baseline disease severity and inhaled corticosteroid (ICS) coverage. Adjusted for age, sex, comorbidities, proportion of days covered by ICS and exacerbation history (number of exacerbations) during the baseline period. GINA: Global Initiative for Asthma.

Risk of severe exacerbation in the first year of the study period was higher in patients who overused SABA when adjusted for age, sex and baseline comorbidities (NS: IRR 1.36, 95% CI 1.18–1.56; AB: IRR 1.32, 95% CI 1.27–1.38; figure 5). The effect estimates were consistent between provinces (albeit nonsignificant in NS) among patients with high utilisation of ICS-containing medication (six or more canisters) and when stratified by disease severity (GINA Steps). These effect estimates also remained within bounds of the main analysis, albeit with larger confidence intervals associated with smaller sample size.

## Discussion

More than 25% of patients with mild asthma were observed to have SABA overuse in the Canadian provinces of NS and AB. SABA overuse was associated with an increased rate of severe exacerbations and this association remained when stratified by ICS use (six or more canisters of ICS per year). The results of the present study are consistent with prior Canadian studies characterising SABA use nearly two decades ago [7] and those describing an association with higher risk of mortality and other poor health outcomes [21, 24, 30]. FitzGerald *et al*. [7] identified individuals who used two or more doses of SABA per week in the absence of any ICS or used more than nine canisters of SABA during the year and no more than 100 μg·day^−1^ of ICS. They found that inappropriate SABA use was associated with a 45% increased risk of hospital admissions and a 25% increased risk of emergency department visits in the 3-month period following that inappropriate use [7]. In addition, a recent Canadian study including patients ≥65 years of age with prevalent asthma found 14% of patients to have overused SABA (three or more canisters per year) and an associated increased risk of severe asthma exacerbations, ranging from 59–126% among those using three to five canisters and six or more canisters per year [24]. Thus, while definitions of SABA overuse vary in specificity [38], the association between overuse of SABA and poor health outcomes is consistent with the results found in our study.

Internationally, SABA overuse varies from 10% to ∼40% depending on study design [16, 17, 39]. Results from the SABINA participating countries of similar health systems using claims data were comparable to those found in this study [16, 17]. In these countries, the rate of SABA overuse in a real-world setting ranged from 32% (Italy) to 38% (UK), and was associated with a corresponding increased risk of exacerbations of 27% (Italy) and 41% (UK) [16, 17, 40]. While differences in access, policies, healthcare and/or environment may contribute to differences in outcomes, this study observed a rate of SABA overuse ranging from 28% (AB) to 39% (NS) and an estimated adjusted increased risk of severe exacerbations ranging from 32% (AB) to 36% (NS). As such, this study contributes to the body of evidence and supports a common finding of increased risk of exacerbation associated with SABA overuse [38].

The availability of comprehensive population-based data is a key strength of this study, adding to the generalisability of our results across Canada. In addition, asthma-related prescriptions are comprehensively captured in these data, because SABA can only be dispensed *via* prescription in Canada. Nonetheless, the use of administrative claims data in this context is also associated with several limitations. Prescription dispensation does not confirm actual use (and the timing of use) of study medications, *e.g.* patients may acquire new inhalers prior to fully utilising prior inhalers. As a result, while associations between SABA overuse and exacerbation outcomes were estimated in this study, causal inference of this relationship is not possible. In addition, claims data do not present additional clinical detail such as a measure of asthma control. As a result, medication and health resource use were used to characterise outcomes and baseline disease severity. Similarly, the dispensation of OCS used to define exacerbations would be subject to the same limitations. Therefore, in the absence of symptom or disease scores, the disease characteristics of these included patients is largely based on dispensed medication rather than actual use and clinical evaluation.

### Implications for practice

Several randomised trials have already detailed a reduced risk of severe exacerbations in patients receiving budesonide/formoterol as needed compared to SABA alone, highlighting ICS/LABA as an advantageous combination relative to SABA in patients with mild asthma [41–45]. Among the population of very mild and mild asthma patients, it is now recommended by both GINA and CTS guidelines that patients with poorly controlled asthma or well-controlled asthma at a high risk of exacerbation should be started on daily ICS with as-needed SABA, or budesonide/formoterol as needed [34, 46]. In our study, we found that this guidance would imply an immediate change in treatment strategy for 28% of the AB and 39% of the NS cohort, *i.e.* those patients who were overusing SABA to manage their asthma symptoms and who were at increased risk of severe exacerbation. As a result, these findings support the recent CTS guidelines for mild to moderate asthma: 1) patients with very mild and mild asthma may still be at risk of severe exacerbations and their disease control should be regularly assessed; 2) SABA use can be used as an indicator of partially controlled or uncontrolled asthma and three or more canisters of SABA in a year is considered overuse; 3) overuse of SABA is associated with a higher risk of exacerbation.

These findings may also have implications for patients with moderate to severe asthma. For these patients, the CTS stratifies by risk of exacerbation and recommends escalation of ICS dose and/or consideration of biological therapy where appropriate. SABA use in these patients may provide a simple indicator with which to estimate asthma control. Indeed, the CTS now includes three or more doses of SABA per week as indicator of partial/uncontrolled asthma.

Primary care providers and specialists may consider implementing these findings by reviewing with their patients the number of doses of SABA used in a week as a simple and objective way to identify patients who have partially controlled or uncontrolled asthma. This approach has also been highlighted by the Health Quality Ontario updated asthma quality standard, which recommends assessing SABA use at least annually with a recommended cut-off of three or more canisters [47].

### SABA use is a call to action

Physicians should regularly review SABA use with patients, regardless of disease severity. Pharmacists have a valuable opportunity to identify potential SABA overuse, provide education to patients and offer feedback to prescribing clinicians. Patients should be made aware of the implications of SABA overuse and use this simple metric to help manage their disease and advocate for better care.

### Conclusion

More than 25% of patients in this study overused SABA medication during the study period. SABA overuse was associated with increased risk of severe exacerbation outcomes. These findings support the recent updates to the CTS guidelines for asthma care [46], the National Heart, Lung, and Blood Institute and GINA recommendations [48] and the Health Quality Ontario asthma quality standard [47]. Weekly or annual estimates of SABA use represent a simple metric for loss of disease control to physicians, pharmacists and patients and should be considered a simple call to action for all stakeholders.

## Supplementary material

10.1183/23120541.00140-2022.Supp1 **Please note:** supplementary material is not edited by the Editorial Office, and is uploaded as it has been supplied by the author.
Supplementary material 00140-2022.SUPPLEMENT
Figure S1 00140-2022.FIGURE1
Figure S2 00140-2022.FIGURE2
