# Supplementary material for: SABA use as an indicator for asthma exacerbation risk: an observational cohort study (SABINA Canada)
Source: ERJ Open Res. 2022 Sep 26;8(3):00140-2022. doi: 10.1183/23120541.00140-2022 (PMC9511146; doi:10.1183/23120541.00140-2022)
Supplement: Supplementary file 1 [file 00140-2022.SUPPLEMENT.pdf]

Supplementary Appendix

Methods

Defining asthma severity

Using the CTS definitions for ICS prescription, patients were considered to have mild asthma with a low daily dose of up to 250mcg of beclomethasone equivalent. Moderate disease was defined as having a medium daily dose of between 251 and 500 mcg of beclomethasone, or equivalent. Severe asthma was defined as having a high daily dose of >500 mcg of beclomethasone or equivalent. Similar methodology was used to define the GINA steps, with medication types also taken into consideration: Step 1 included patients with no controller treatment, Step 2 included patients with low dose ICS product, leukotriene receptor antagonist (LTRA), or low-dose theophylline alone, Step 3 included patients using low dose combination product of ICS with LABA or LTRA and/or theophylline, or medium/high dose of ICS alone; Step 4 included patients with medium/high dose for combination product of ICS with LABA and/or LTRA, theophylline, and/or tiotropium; and Step 5 included the patients treated with maintenance OCS (excluding OCS use of ≤10 days) with or without any other inhalers were included in Step 5.

Cleaning dispensed prescription claims data for SABA use

For each prescription claim:

- The drug identification number (DIN) was used to obtain package size from the product monograph
- The dispensed quantity was used to divide the package size
- If the quotient is a whole number, it was then assumed that the unit was inhalations
- If the quotient is not a whole number, and dispensed prescription claim has an attached unit indicating it was a canister, it was then assumed that the units were indeed canisters and multiplied by package size to get total number of inhalations
- If the quotient is not a whole number and dispensed prescription claim has an attached unit indicating it was inhalations, it was then assumed that the units were indeed inhalations
- If the quotient is not a whole number and dispensed prescription claim did not have a clear unit attached, then the prescriptions claims with a quantity <30 were assumed to be in units of canisters, and for those above that threshold they were considered as number of inhalations

All of the number of inhalations were tallied up and divided by 150 to get the total number of canisters per year.

Results

Mortality:

In NS, 0.8% of the overall cohort died during the study period. This ranged from 0.6% among mild patients (excluding those without prescription) to 2.6% among severe patients. Mortality among patients in AB was lower, with a mortality rate of 0.07% among the overall cohort. Mortality in AB was predominately observed among patients with severe disease (0.14%), followed by patients with no prescription of any severity at baseline (0.09%); mild and moderate asthma patients in AB had similar mortality rates of 0.06% and 0.05%, respectively.

Supplementary Table S1: Anatomical therapeutic chemical (ATC) classification codes and drug identification numbers (DINs) of commonly used ICS and SABAs in Canada.

	Generic Name	ATC/DDD	DIN(s)
ICS	**Beclomethasone**	R03BA01<br>R03AK08	02242029, 02242030
	**Fluticasone propionate***	R03BA05<br>R03AK06<br>R03AK11	02467895, 02467909, 02467917, 02237245, 02237246, 02237247, 02244291, 02244292, 02244293<br>02240835, 02240836, 02240837, 02245126, 02245127, 02474611, 02474638, 02474646
	**Budesonide***	R03BA02<br>R03AK07<br>R03AK12	02229099, 01978918, 01978926, 00851752, 00851760, 00852074, 02465949, 02465957<br>02245385, 02245386, 02248218
	**Ciclesonide**	R03BA08	02285606, 02285614
	**Mometasone**	R03BA07<br>R03AK09	02243595, 02243596, 02438690<br>02361744, 02361752, 02361760
	**Fluticasone furoate***	R03BA09<br>R03AK10	02446561, 02446588<br>02408872, 02444186
SABA	**Salbutamol**	R03AC02	02469359, 02232570, 02245669, 02208229, 02208237, 02208245, 02419858, 02326450, 01926934, 02173360, 02243115, 02241497, 02213419, 02213427, 02213486
	**Terbutaline**	R03AC03	00786616
***include ICS/LABAs**			

Supplementary Table S2: Oral corticosteroid and biologics drug codes

	Generic Name	ATC/DDD	DIN(s)
**Oral corticosteroids**	Prednisone	H02AB07	00550957, 00312770, 00021695, 00232378, 00271373
	Prednisolone	H02AB06	02230619,02245532
	Methylprednisolone	H02AB04	00036129, 00030988
	Dexamethasone	H02AB02	02239534, 02250055, 02261081, 01946897, 01964976, 01964968, 01964070, 02279363
	Hydrocortisone	H02AB09	00030910, 00030929
	Cortisone	H02AB10	00280437
**Biologics**	Omalizumab	R03DX05	2260565, 02459787, 02459795
	Mepolizumab	R03DX09	02449781
	Reslizumab	R03DX08	02456419
	Benralizumab	R03DX10	02473232
	Dupilumab	D11AH05	02470365

Supplementary Table S1 Outcome identifying codes

		Code type	Codes
Exacerbation	Short course oral corticosteroids	ATC/DDD	H02AB07, H02AB06, H02AB04, H02AB01, H02AB02, H02AB09, H02AB10
		DINs	00550957, 00312770, 00021695, 00232378, 00271373<br>02230619,02245532<br>01934325, 01934333, 01934341, 00030759, 00030767, 00036129, 00030988, 02231893, 02231895, 02241229, 00030678, 00036137, 02367947, 02367955, 02367963, 02367971<br>00028096<br>02250055, 02261081, 02387743, 00874582, 00664227, 01977547, 02412888, 02412896, 02204266, 02204274, 01946897, 01964976, 01964968, 01964070, 02279363, 00783900<br>00030910, 00030929<br>00280437
	Hospital admission/ Emergency room visits for asthma (primary diagnosis)	ICD-9	493
		ICD-10	J45
Comorbidities	Gastro-oesophageal reflux	ICD-9	530.8
		ICD-10	K21.9
	Anxiety	ICD-9	300
		ICD-10	F41
	Heart failure	ICD-9	428
		ICD-10	I50
	Pulmonary vascular diseases	ICD-9	415-417
		ICD-10	I26-I28
	Pneumonia	ICD-9	480-487.0
		ICD-10	J10.0, J11.0, J12-J18
	Ischemic heart disease	ICD-9	410-414
		ICD-10	I20-I25
	Arrhythmia	ICD-9	427
		ICD-10	I47-I49
	Myocardial infarction	ICD-9	410
		ICD-10	I21
	Angina	ICD-9	413
		ICD-10	I20
	Diabetes	ICD-9	250.x0, 250.x2
		ICD-10	E11
	Bone fractures	ICD-9	800-829
		ICD-10	S02, S12, S22, S32, S42, S52, S62, S72, S82, S92
	Chronic kidney disease	ICD-9	585
		ICD-10	N18
	Osteoporosis	ICD-9	733.0
		ICD-10	M81
	Depression	ICD-9	296.2, 296.3
		ICD-10	F32, F33
	Anemia	ICD-9	280, 281, 285.9
		ICD-10	D50-53, D64.9

Supplementary Table S2 Individual comorbidities at baseline

	Nova Scotia (N=8,034)	Alberta (N=107,444)
**Asthma-related conditions, n(%)**		
Gastroesophageal reflux	388 (4.2)	958 (0.9)
Anxiety	1,298 (16.2)	29,699 (27.6)
**Other co-morbid conditions, n(%)**		
Heart failure	55 (0.7)	915 (0.9)
Pulmonary vascular diseases	25 (0.3)	732 (0.7)
Pneumonia	351 (4.4)	4,839 (4.5)
Ischemic heart disease	242 (3.0)	4,105 (3.8)
Arrhythmia	139 (1.7)	4,130 (3.8)
Myocardial infarction	30 (0.4)	421 (0.4)
Angina	41 (0.5)	2,113 (2.0)
Diabetes	602 (7.5)	1,981 (1.8)
Bone fractures	192 (2.4)	7,111 (6.6)
Chronic kidney disease	45 (0.6)	958 (0.9)
Osteoporosis	70 (0.9)	958 (0.9)
Depression	59 (0.7)	958 (0.9)
Anemia	303 (3.8)	4,038 (3.8)

Supplementary Table S3 ICS and SABA use by baseline disease severity (GINA steps) during study period

	Nova Scotia Baseline asthma severity (GINA)					Alberta Baseline asthma severity (GINA)				
	**Step 1** (N=2,642)	**Step 2** (N=1,541)	**Step 3** (N=2,453)	**Step 4** (N=1,049)	**Step 5** (N=349)	**Step 1** (N=25,396)	**Step 2** (N=16,472)	**Step 3** (N=39,367)	**Step 4** (N=11,835)	**Step 5** (N=14,374)
**ICS use, n(%)**										
No ICS	2,148 (81.3)	583 (37.8)	426 (17.4)	15 (1.4)	129 (37.0)	17,747 (69.9)	3,685 (22.4)	4,760 (12.1)	157 (1.3)	3,007 (20.9)
Reduced	-	-	84 (3.4)	366 (34.9)	34 (9.7)	-	-	887 (2.3)	5,436 (45.9)	1,237 (8.6)
Stable	477 (18.1)	894 (58.0)	1,714 (69.9)	628 (59.9)	165 (47.3)	7,505 (29.6)	12,156 (73.8)	31,154 (79.1)	6,018 (50.8)	9,317 (64.8)
Increased	17 (0.6)	64 (4.2)	229 (9.3)	40 (3.8)	21 (6.0)	144 (0.6)	631 (3.8)	2,611 (6.6)	381 (3.2)	851 (5.9)
**Annual SABA use, n(%)**										
0	904 (34.2)	432 (28.0)	902 (36.8)	293 (27.9)	110 (31.5)	4,009 (15.8)	2,569 (15.6)	10,276 (26.1)	3,057 (25.8)	2,445 (17.0)
1-2	704 (26.6)	506 (26.8)	673 (27.5)	254 (24.3)	89 (25.5)	14,915 (58.7)	9,695 (58.9)	18,878 (48.0)	4,507 (38.1)	7,061 (49.1)
3+	1,034 (39.1)	603 (39.1)	878 (35.8)	502 (47.9)	150 (43.0)	6,472 (25.5)	4,208 (25.5)	10,213 (25.9)	4,271 (36.1)	4,868 (33.9)
12+	336 (12.7)	175 (11.4)	223 (9.1)	173 (16.5)	54 (15.5)	848 (3.3)	397 (2.4)	1,026 (2.6)	597 (5.0)	756 (5.3)

ICS: inhaled corticosteroids; SABA: short-acting beta agonists
*excluding patients with no prescription for ICS at baseline
Note that those with 'reduced' ICS are those that had moved down an ICS dose category (low, medium, high), whereas those with 'increased' ICS are those that moved up a category, and stable are those who remained in the same category.

Supplementary Table S4 Severe exacerbations by baseline disease severity (GINA steps) during study period

	Nova Scotia					Alberta				
**Annual exacerbations*** *n(%)*	**Step 1** (N=2,642)	**Step 2** (N=1,541)	**Step 3** (N=2,453)	**Step 4** (N=1,049)	**Step 5** (N=349)	**Step 1** (N=25,396)	**Step 2** (N=16,472)	**Step 3** (N=39,367)	**Step 4** (N=11,835)	**Step 5** (N=14,374)
<1	2,483 (94)	1430 (92.8)	2,257 (92)	919 (87.6)	181 (51.9)	24,336 (95.8)	15,757 (95.7)	37,182 (94.4)	11,061 (93.5)	10,661 (74.2)
1 to 2	99 (3.7)	70 (4.5)	120 (4.9)	66 (6.3)	36 (10.3)	881 (3.5)	595 (3.6)	1,791 (4.5)	627 (5.3)	2,221 (15.5)
2+	60 (2.3)	41 (2.7)	76 (3.1)	64 (6.1)	132 (37.8)	179 (0.7)	120 (0.7)	394 (1.0)	147 (1.2)	1,492 (10.4)
**Annual exacerbations,** *mean rate per patient (SD)*	0.22 (0.7)	0.23 (0.7)	0.27 (0.71)	0.50 (2.33)	2.16 (2.82)	0.12 (0.36)	0.12 (0.37)	0.14 (0.41)	0.16 (0.45)	0.70 (1.53)
