# Supplementary material for: SABA use as an indicator for asthma exacerbation risk: an observational cohort study (SABINA Canada)
Source: ERJ Open Res. 2022 Sep 26;8(3):00140-2022. doi: 10.1183/23120541.00140-2022 (PMC9511146; doi:10.1183/23120541.00140-2022)
Supplement: Supplementary file 2 [file 00140-2022.FIGURE1.pdf]

Appendix Figure 1 Study design diagram

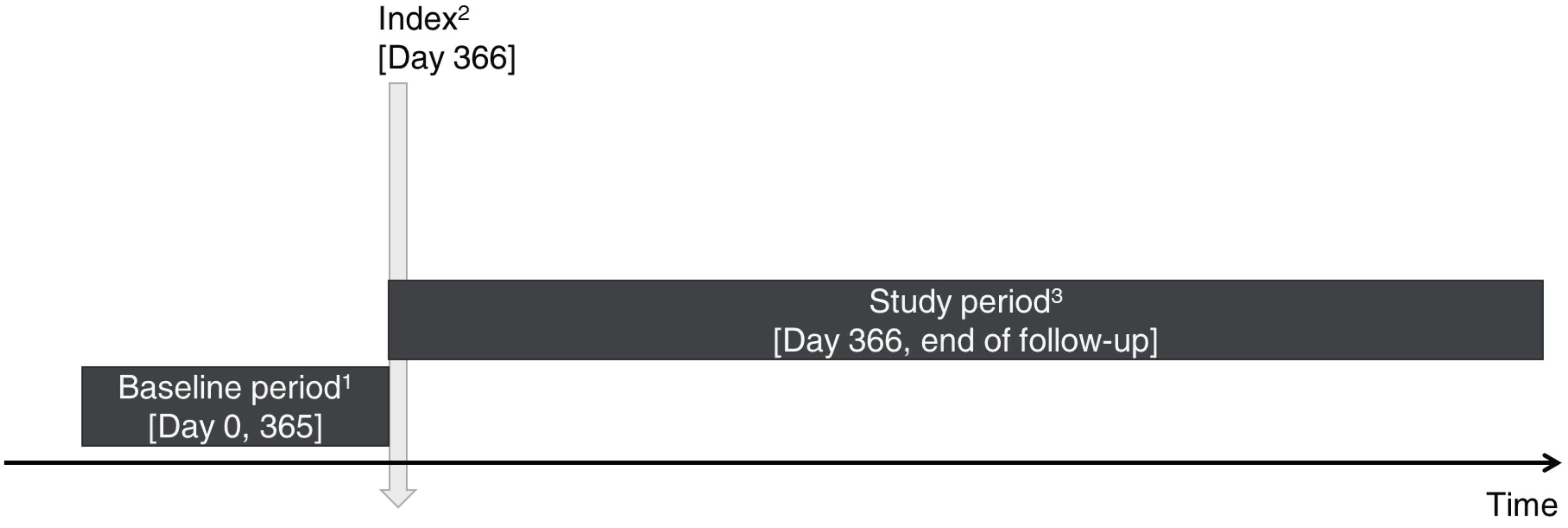

1. Used to assess case definition and to characterize baseline comorbidity, asthma disease severity, the frequency of asthma-related prescription claims, physician visits, and exacerbations; note that baseline period started at the first eligible diagnosis meeting the case definition of ≥2 physician visits in a two-year period or ≥1 hospital separation admission with a diagnosis of asthma [ICD-9-CM 493 or ICD-10-CA J45], with the earliest date possible being October 1st, 2016 for Nova Scotia and April 1st, 2016 for Alberta
2. Used to characterize the age and sex of patients included
3. Used to measure all study outcomes; note that end of follow-up was defined as the first occurrence of death, emigration from the province, or the end of the study period (Nova Scotia: March 31st 2019; Alberta: March 31st, 2020)
