# Supplementary figures and images for: SABA use as an indicator for asthma exacerbation risk: an observational cohort study (SABINA Canada)
Source: ERJ Open Res. 2022 Sep 26;8(3):00140-2022. doi: 10.1183/23120541.00140-2022 (PMC9511146; doi:10.1183/23120541.00140-2022)

Appendix Figure 2 Study consort diagram

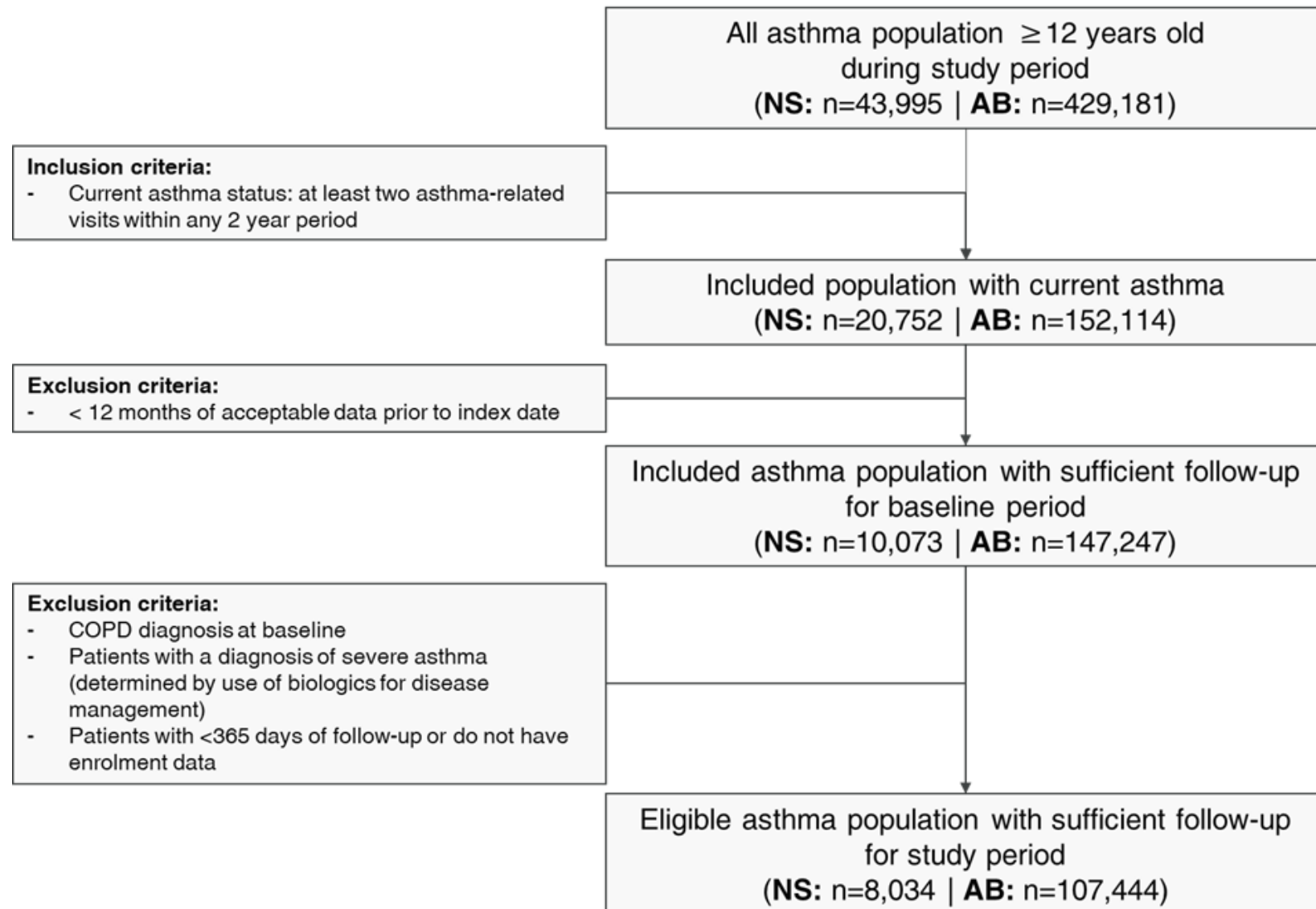

Supplement: Supplementary file 3 [file 00140-2022.FIGURE2.pdf]
